# Feeding behaviour of potential vectors of West Nile virus in Senegal

**DOI:** 10.1186/1756-3305-4-99

**Published:** 2011-06-08

**Authors:** Assane G Fall, Amadou Diaïté, Renaud Lancelot, Annelise Tran, Valérie Soti, Eric Etter, Lassana Konaté, Ousmane Faye, Jérémy Bouyer

**Affiliations:** 1 Institut Sénégalais de Recherches Agricoles/Laboratoire National de l'Elevage et de Recherches Vétérinaires BP 2057 Dakar-Hann, Sénégal; 2 Centre International en Recherche Agronomique pour le Développement (CIRAD), UMR CIRAD-INRA Contrôle des maladies animales exotiques et émergentes, Campus international de Baillarguet, 34398 Montpellier Cedex 5, France; 3 CIRAD, UMR TETIS, Maison de la Télédétection, 34093 Montpellier Cedex 5, France; 4 CIRAD, UR Animal et Gestion Intégrée des Risques (AGIRs), 34398 Montpellier Cedex 5, France; 5 Département de Biologie Animale, Faculté des Sciences et Techniques, Université Cheikh Anta Diop, Dakar, Sénégal; 6 Department Environment and Societies, TREP Building, University of Zimbabwe, P.O. Box 1378, Harare, Zimbabwe

## Abstract

**Background:**

West Nile virus (WNV) is a widespread pathogen maintained in an enzootic cycle between mosquitoes and birds with occasional spill-over into dead-end hosts such as horses and humans. Migratory birds are believed to play an important role in its dissemination from and to the Palaearctic area, as well as its local dispersion between wintering sites. The Djoudj Park, located in Senegal, is a major wintering site for birds migrating from Europe during the study period (Sept. 2008- Jan. 2009). In this work, we studied the seasonal feeding behaviour dynamics of the potential WNV mosquito vectors at the border of the Djoudj Park, using a reference trapping method (CDC light CO_2_-baited traps) and two host-specific methods (horse- and pigeon-baited traps). Blood meals of engorged females were analysed to determine their origin.

**Results:**

Results indicated that *Culex tritaeniorhynchus *and *Cx. neavei *may play a key role in the WNV transmission dynamics, the latter being the best candidate bridging-vector species between mammals and birds. Moreover, the attractiveness of pigeon- and horse-baited traps for *Cx. neavei *and *Cx. tritaeniorhynchus *varied with time. Finally, *Cx. tritaeniorhynchus *was only active when the night temperature was above 20°C, whereas *Cx. neavei *was active throughout the observation period.

**Conclusions:**

*Cx. neavei *and *Cx. tritaeniorhynchus *are the main candidate vectors for the transmission of WNV in the area. The changes in host attractiveness might be related to variable densities of the migratory birds during the trapping period. We discuss the importance of these results on the risk of WNV transmission in horses and humans.

## Background

West Nile fever (WNF) is an arthropod-borne disease caused by a *Flavivirus *(Flaviviridae) belonging to the Japanese encephalitis antigenic complex [1]. Birds are involved in its pathosystem. Horses and humans are dead-end hosts: their infection often remains unapparent but they can suffer febrile or even fatal illness with neural symptoms [2]. The West Nile virus (WNV) is highly endemic in Africa in general, and particularly in Senegal [3-5]. Migratory birds may be involved in spreading the virus in Africa, Europe, the Middle East and south-western Asia, especially through the Palaearctic migration routes where major flyways are crossing each other [6]. Such transcontinental introduction must be anchored to local (African) spreading mechanism especially at birds' nesting, feeding, or resting sites where vector feeding behaviour probably plays a critical role [7].

The Senegal River delta (northern Senegal and southern Mauritania) is characterized by a mixture of natural wetlands and extensive irrigated agricultural activity. It is one of the major wintering sites for birds migrating between Europe and Africa that benefit from abundant food resources in the Djoudj National Park, where this study was conducted. The majority of the bird species migrating from Europe arrive there during the month of October and start their return flight during March/April [8].

A number of mosquito candidate vectors for the transmission of WNV have been identified in Senegal: *Culex poicilipes, Cx. naevei, Mymomia spp., Mymomia hispida, M. lacustris, M. splendens, Aedomya africana *[5], *A. vexans *and *Mansonia uniformis *[9]. All these species have a nocturnal feeding behaviour. However, little is known regarding their host preferences, especially regarding species feeding both on birds and mammals. The main objective of this study was to assess their feeding behaviour and its seasonality during the period of high risk of transmission of WNV in the area [3], to identify potential factors increasing the transmission between birds, and more importantly, from birds to mammals.

**Figure 1:**
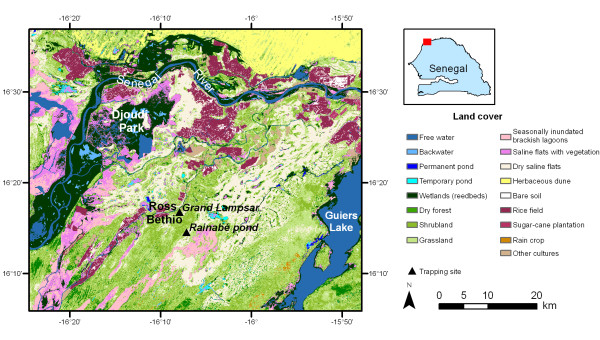
**Ross Bethio study area in the delta of the Senegal River**. The mosquito trapping sites are projected on the land cover (data source: EDEN project, http://www.eden-fp6project.net/).

The study was conducted in Ross Bethio, a small town located 10 km south from the Djoudj National Park, Senegal (Figure 1). In 2005, a serological survey carried out on horses in this region highlighted high WN prevalence rates (0.85; n = 367; 95% CI 0.81-0.89) [10].

The landscape surrounding Ross-Bethio is characterized by grasslands, shrublands and dry saline flats ("tans"). This area is also one of the main agricultural centres in the Senegal River delta, rice and sugar cane representing the main crops. During the dry season (from November to May), green vegetation is limited to some scattered trees and shrubs. During the rainy season (June to October: mean annual rainfall ca. 250 mm), an herbaceous layer is available for domestic ruminants, donkeys and horses. The latter is used for ploughing, transporting goods and humans, and social distinction. The Senegal River valley, including the delta, is flooded each year either by rainfall water naturally collected in the upper Senegal River basin, or by planned water releases from the Manantali dam (upper Senegal River, Mali) while retaining water with the Diama dam located near Ross Bethio. In Ross Bethio, the maximum flooding level is usually observed in early November.

## Results

In total, 28,965 female mosquitoes, representing 12 species in 5 genera, were captured in all traps over 79 nights from September 2008 to January 2009. Mosquitoes from the *Culex *genus represented 95% (27,443) of the total captures. The predominant species were *Cx. tritaeniorhynchus, Cx. neavei *and *Cx. poicilipes*, representing 69.4% (20,109), 19.7% (5,702) and 5.4% (1,575) of the total captures respectively. CDC light CO_2_-baited traps were the most efficient with 20,382 female mosquitoes (apparent nightly density per trap - ANT of the total collection = 566.2) in 11 species belonging to 5 genera closely followed by the horse-baited trap with 7402 female mosquitoes (ANT of the total collection = 493.5) in 8 species and 4 genera. Pigeon-baited traps collected 1,181 (ANT of the total collection = 42.2) mosquitoes in 6 species and 2 genera (Table 1).

**Table 1:** Mean apparent mosquito densities in the Senegal River delta

	CDC light CO2-baited traps			Horse-baited trap	Pigeon-baited traps	
	
Mosquito species	GL ground (n = 15)	GL canopy (n = 12)	RM ground (n = 9)	RB (n = 15)	GL ground (n = 14)	GL canopy (n = 14)
*Culex neavei*	53.7 ± 53.41	106.3 ± 121.51	10.4 ± 21.54	176.7 ± 275.47	3.3 ± 4.16	59.4 ± 38.82
*Culex tritaeniorynchus*	540.3 ± 755.92	408.1 ± 674.02	331.5 ± 540.06	257.4 ± 485.02	1.6 ± 3.71	17.4 ± 33.36
*Aedes sudanensis*	0	0.1 ± 0.29	0.7 ± 1.12	0	0	0
*Mansonia uniformis*	21.2 ± 21.92	18.3 ± 24.50	5 ± 5.96	2.2 ± 3.23	0.4 ± 0.74	0.5 ± 0.85
*Aedomyia africana*	0.1 ± 0.35	2.1 ± 2.75	0	0.1 ± 0.26	0	0
*Anopheles rufipes*	0	0.1 ± 0.29	0.1 ± 0.33	0.1 ± 0.26	0	0
*Anopheles pharoensis*	6.3 ± 7.82	3.2 ± 3.54	4.8 ± 8.67	29.8 ± 44.25	0	0
*Anopheles ziemanni*	4.3 ± 7.50	3.9 ± 6.72	1.8 ± 2.49	7.1 ± 8.67	0	0
*Culex bitaeniorhynchus*	0.9 ± 1.39	0.2 ± 0.39	2 ± 4.69	0	0	0.2 ± 0.43
*Culex theileri*	0	0	0	0	0	0.1 ± 0.27
*Culex poicilipes*	16.3 ± 15.96	68 ± 89.94	21.2 ± 49.73	20.1 ± 26.33	0	1.6 ± 2.82
*Culex perfuscus*	0.7 ± 1.39	0.3 ± 0.89	0.1 ± 0.33	0	0	0

Mean values per trap per night ± standard deviation for each mosquito species during the whole trapping period are presented according to the trap type, height and location (RB = Ross Bethio, GL = Grand Lampsar, RM = Raïnabé tempory pound, n = number of trap-nights).

The predominance of *Cx. tritaeniorhynchus *and *Cx. neavei *over the other species was also clearly showed on the table 1. The ANTs within a given trap type were correlated between sites (*p *< 0.05). Whereas *Cx. tritaeniorhynchus *was mainly mammophilic, *Cx. neavei *was attracted by both horses and birds.

**Figure 2:**
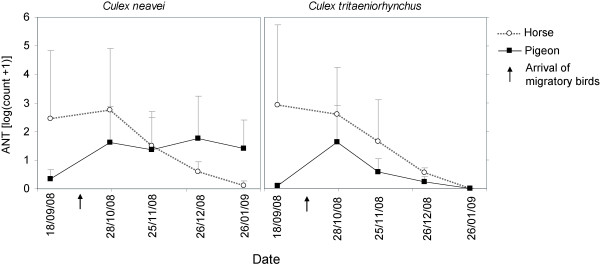
**Seasonal variations of mosquito densities in the Senegal River delta (Senegal)**. The mean apparent mosquito densities per trap per night are presented for *Culex neavei *and *Cx. tritaeniorhynchus *in horse and pigeon-baited traps (standard errors are presented as vertical lines).

A strong seasonality was observed in the attractiveness of each host for *Cx. neavei *and *Cx. tritaeniorhynchus*. The ANTs of *Cx. neavei *and *Cx. tritaeniorhynchus *were highest in September and October in the horse-baited trap and then decreased until January. In pigeon-baited traps, they increased in October for both species and remained stable for *Cx. neavei *whereas they decreased again until January for *Cx. tritaeniorhynchus *(Figure 2). In October, both mosquito species fed on horses and birds.

**Figure 3:**
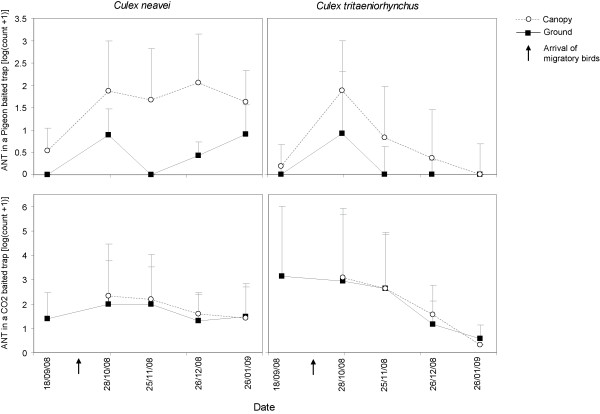
**Seasonal variations of mosquito densities according to trap location at Grand Lampsar River, in the Senegal River delta**. The mean apparent mosquito densities per trap per night are presented for *Culex tritaeniorhynchus *and *Cx. neavei *according to the location of the pigeon- and CO_2_- baited traps, on the ground or in the tree canopy (standard errors are presented as vertical lines).

The height of the trap had an impact on host attractiveness. In the CDC light CO_2_-baited traps, the ANTs of the two predominant species (*Cx. tritaeniorhynchus *and *Cx*. *neavei) *did not differ significantly when placed at the ground or in the tree canopy in Grand Lampsar (*p *> 0.05). On the other hand, these ANTs differed significantly between heights in pigeon-baited traps for *Cx. tritaeniorhynchus *(*p *= 0.02), and *Cx. neavei *(*p *= 4 × 10^-5^). These differences were significant only after October (Figure 3). Whereas the ANTs in the CO_2_-baited CDC traps decreased during the study both in the tree canopy and on the ground (as with the horse-baited traps), they increased sharply in October for both species in the pigeon-baited traps, particularly in the tree canopy, and remained stable for *Cx. neavei *until the end of the study period, whereas they decreased again in *Cx. tritaeniorhynchus*.

Most of the engorged mosquitoes were collected from the CO_2_-baited traps placed on the ground (38 out of 45). Blood meals analyses (Table 2) revealed that *Cx. neavei *fed mainly on birds, followed by humans, cattle and horses. *Culex tritaeniorhynchus *blood meals were taken on horses, humans, cattle, and birds. Mixed meals identified from *Cx. tritaeniorhynchus *(14% of total blood meals) were associations between sheep and cattle (50%), human and cattle (25%) and human and horse (25%).

**Table 2:** Origin of mosquito bloodmeals in the Senegal River delta

		Vertebrate hosts (%)							
			
Mosquito species	Total number of samples	Horse	Cattle	Birds	Human	Cattle, Sheep	Human, Cattle	Human, Horse	Not identified blood meals (%)
*Cx. neavei*	14	1(7.1)	2(14.3)	4(28.6)	3(21.4)	0(0.0)	0(0.0)	0(0.0)	4(28.6)
*Cx. tritaeniorhynchus*	28	10(35.7)	4(14.3)	1(3.6)	5(17.8)	2(7.1)	1(3.6)	1(3.6)	4(14.3)

The results are presented for *Culex neavei *and *Cx. tritaeniorhynchus *captured in CO2-baited CDC traps (percentage in brackets).

**Figure 4:**
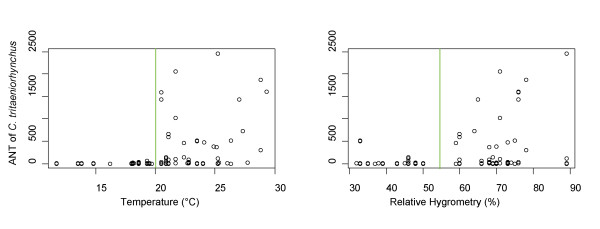
**Impact of temperature and relative hygrometry on the density of mosquitoes in the Senegal River delta**. The panels present the relationship between the mean apparent mosquito densities per trap per night of *Culex neavei *and *Cx. tritaeniorhynchus *and these meteorological parameters.

There was no relationship either between the temperature or the relative hygrometry and the ANTs of *Cx. neavei *(*p *= 0.82). Conversely, the ANTs of *Cx. tritaeniorhynchus *were correlated with temperature (*p *= 0.001) and relative hygrometry (*p *= 7 × 10^-9^). Indeed, the activity of *Cx. tritaeniorhynchus *was very low when temperature was below 20°C and\or the relative hygrometry was below 55% (Figure 4).

## Discussion

Greater mosquito numbers and higher species diversity were caught in CO_2_-baited CDC light traps, as compared with the other traps. CO_2_-baited CDC light traps allow a representative census of the mosquito diversity in a given area, particularly arbovirus vectors in Senegal [5,11,12]. All captured mosquito species, but *Cx. theileri*, were recorded in these traps in Ross Béthio. Indeed, the latter seems to be very rare in the area. Only 45 females (0.2%) were found engorged. Such a low proportion has been observed in other studies [13]: indeed, starving mosquitoes are more attracted by CO_2_-baited traps (which mimic hosts' breath) than engorged mosquitoes.

*Culex poicilipes, Cx. tritaeniorhynchus, Cx. neavei*, and *Mansonia uniformis *were the most abundant mosquito species given the traps used. In Senegal, WNV has been isolated, or WN viral ribonucleic acid has been detected, from each of these species [5,12,14]. The competence of *Cx. tritaeniorhynchus *and *Cx. neavei *for the WNV has been demonstrated [15,16]. These two species are thus possible WNV vectors in this area. Regarding other mosquito species, virus isolations do not imply that these species are competent WNV vectors. It is only an indication that they had fed on viremic hosts.

Chevalier *et al. *[7] found higher prevalence rate (*p *= 3 × 10^-4^) for WNV in resident birds resting on the ground (0.10, *n *= 88) than in those resting in the bush (0.04, *n = *125) or in the canopy (0.04, *n=*209). In this study, pigeon baited-traps captured a lower diversity of mosquito species (5 *Culex *species and 1 *Mansonia*). The height of the pigeon baited-trap had an effect on its attractiveness for *Cx. tritaeniorhynchus *and *Cx. neavei*, conversely to the CO_2_-baited trap. However, this difference depended on the bait and the season, and was only noticeable from October, when the migratory birds are known to arrive from Palaearctic areas for wintering [17]. We also observed a shift of feeding hosts of *Cx. neavei *and *Cx*. *tritaeniorhynchus *starting in October, from horses to birds. However, the dataset is limited and the observed shift difficult to explain. Our main hypothesis is that they were caused by changes in host availability combined with host and site fidelity in these two mosquito species: the night resting sites of passerine birds is the tree canopy, and their increase in abundance might increase the attractiveness of pigeon-baited traps set in the tree canopy. Actually, host fidelity has been reported in other vector species [18,19] while site fidelity has been reported in anophelines [20]. Host fidelity was also reported in experimental conditions for *Cx. tritaeniorhynchus *[21]. Mark-release-recapture experiments also suggested that two other *Culex *species might "memorize" flight paths within their environment [22]. In tsetse flies, the decomposition of habitats into a "home range" and a "feeding ground" - between which insects would fly on purpose [23], was recently assessed using population genetics [24]. On the contrary, in opportunistic mosquitoes such as *Aedes vexans arabiensis*, the feeding behaviour is linked to host availability [25]. Unfortunately, we could not validate these shifts in feeding patterns using the ELISA results because of limited sample size (only 45 blood-fed mosquitoes). This study should thus be repeated, including a follow up of the resting sites and abundance of birds from different orders (Passeriformes, Galliformes, Charadriiformes, etc.), and continued during the departure of migratory birds in March to April to confirm that the mosquitoes would go back to the available mammal hosts. In the Ferlo region of Senegal, 175 km south-east from Ross Bethio, *Cx. neavei *was also found more abundant in the tree canopy than on the ground, using both pigeon- and chicken-baited traps [9].

The study period ranged from Sept. 2008 to Jan. 2009, corresponding to the wintering of European bird species in the Djoudj National Park [8,17], as well as to a high density of potential mosquito WN vectors: end of rainy season, flooding of the Senegal River Valley, optimum temperature and relative hygrometry for the development and the survival of vectors. A prospective serological survey carried out during the same time period on a cohort of seronegative horses, showed that WNV circulation occurred mainly between October and January in Ross Bethio (Diouf, pers. com.). The same WNV transmission period was observed in sentinel chicken in the Ferlo [3], also when surface water and mosquitoes were abundant, and the migratory birds arrived in the area [26].

If the shift of feeding patterns were to be confirmed, it might favour the transmission of the WNV from birds to horses. Actually, between-bird WNV transmission [27] would be increased due to a higher ornithophilic tropism in *Cx. neavei *when birds occur at high densities, but then the risk of WNV transmission to horses and humans would increase again with the departure of birds. Results of blood meal analyses confirmed that humans were the second hosts after horses in *Cx. tritaeniorhynchus*, and after birds in *Cx. neavei*. Such shifts from birds to humans were demonstrated for *Culex pipiens *and *Cx. tarsalis *in North America [28]. They were governed by the dispersal of their bird preferred hosts and lead to higher risk of WNV infection in human.

Lastly, the activity of *Cx. tritaeniorhynchus *was governed by a threshold in temperature/hygrometry (Figure 4), which was not observed for *Cx. neavei*.

## Conclusions

Without neglecting the possible role of other mosquito species in the transmission of the WNV, results obtained in this study suggest that *i*) *Cx. neavei *and *Cx. tritaeniorhynchus *are the main candidate vectors for the transmission of WNV, *ii*) *Cx. neavei *is probably a permanent between-bird vector, and a seasonal bridge vector between birds and mammalian hosts, *iii*) *Cx. tritaeniorynchus *is probably a seasonal less important bridge vector between birds and mammalian hosts.

## Methods

### Trapping systems

The Centers for Disease Control and Prevention (CDC) light trap [29] baited with CO_2 _was used as the reference trap. It was compared with animal-baited traps using pigeons and horses. The horse-baited trap was a steel cage (2.5 × 1.5 × 2 m) containing a horse and covered with a mosquito netting (4 × 3.5 × 2.5 m) hanging approximately at 15 cm from the ground, thus allowing the mosquitoes to enter. This model was used by Balenghien et *al*. [30] to study the potential WNV vectors during an outbreak of this disease in Southern France. The pigeon-baited trap was a plastic cylinder of 31.5 cm diameter and 90 cm length divided into 3 compartments. The central compartment was 50 cm long and contained the pigeon box. The two lateral compartments measured each 20 cm and were closed at their sides akin to the central compartment by a mosquito net. Funnel of mosquito netting with an internal opening of about 3 cm of diameter were fixed to the outside sides and allowed the mosquitoes to enter. Darbro and Harrington [31] used this trap model for surveillance of WN mosquito vectors in the New York state (USA). Mosquitoes captured in these animal-baited traps were collected by aspiration.

### Study site

The CDC light CO_2_-baited trap was set near the Grand Lampsar River (16.279°N, 16.132°W), a tributary of the Senegal River and near the Rainabe temporary pond (16.242° N, 16.119° W) (Figure 1). The pigeon baited traps were set only near the Grand Lampsar River. CDC light CO_2_-baited trap and pigeon baited traps were placed either at about 1.5 m high from the ground, or at about 6 m high in the tree canopy, at the extremities of square of about 10 by 10 m. The horse-baited trap was placed in the stable of a horse owner in Ross Bethio (16.268° N, 16.133° W).

Mosquito traps were set overnight from 6 pm to 6 am during three consecutive days, monthly from 16 Sept. 2008 to 28 Jan. 2009. Mosquitoes were identified using the morphological keys of Edwards [32] for the Culicinae subfamily and Diagne *et al. *[33] for the Anophelinae subfamily. Engorged females collected in CDC light CO_2_-baited traps were placed in tubes individually and stored at -20°C until the determination of the origin of their blood meal. The ELISA technique developed by Beier *et al. *[34] was used for these analyses. The choice of conjugates was done taking into account the potential hosts frequenting the trapping sites. The following conjugates were thus used: anti-human, anti-sheep, anti-cattle, anti-chicken, and anti-horse.

Temperature and relative hygrometry data were also collected with the Thermo-Hygro sensor (model NO. THGR228N HUGER^®^) placed in each trapping site. During the 2008 rainy season, the last rains were recorded in the second half of September: no rain was thus recorded during our study.

### Statistical analyses

Trap attractiveness for each mosquito species was computed as the mean number of mosquito individuals from this species in a given trap during three consecutive night catches, also called the mean apparent nightly density per trap (ANT).

A principal component analysis [35] was applied to the whole dataset, with the mosquito species as the individuals and the mean ANTs of each species over the whole period by trap type, height (canopy versus ground) and site as variables, to explore the capture pattern of the various species and the correlations between the trapping systems.

The mean ANTs between sites or heights were compared altogether using a Kruskal-Wallis rank sum test [36], then using a Wilcoxon rank sum test [36].

The R software package was used for statistical analyses [37].

## Competing interests

The authors declare that they have no competing interests.

## Authors' contributions

AGF and AD designed and performed the experiment, and wrote the first draft of the manuscript. AT and VS designed the study area figure and documented it in the draft. AGF, RL and JB designed figures and performed statistical analyses. RL, JB, EE, OF and LK contributed to the manuscript redaction and revised it critically. All authors read and approved the final version of the manuscript.
